# Optimization of Enzymatic Hydrolysis by Protease Produced from *Bacillus subtilis* MTCC 2423 to Improve the Functional Properties of Wheat Gluten Hydrolysates

**DOI:** 10.1155/2024/5053510

**Published:** 2024-06-30

**Authors:** Hari Prasath Rajendhran, Vinoth Kumar Vaidyanathan, Swethaa Venkatraman, Pothiyappan Karthik

**Affiliations:** ^1^ Integrated Bioprocess Laboratory Department of Biotechnology School of Bioengineering SRM Institute of Science and Technology, Kattankulathur, Chennai, Tamil Nadu 603203, India; ^2^ Department of Food Technology Faculty of Engineering Karpagam Academy of Higher Education, Coimbatore 641021, India; ^3^ Centre for Food Nanotechnology Karpagam Academy of Higher Education, Coimbatore 641 021, India

## Abstract

This study is aimed at investigating the reutilizing of gluten protein from the wheat processing industry by *Bacillus subtilis* MTCC 2423 protease to obtain gluten hydrolysates with high added value. Gluten protein hydrolysis using protease achieved a 34.07% degree of hydrolysis with 5% gluten protein, at a hydrolysis time of 2 h for 1000 U/mL at pH 8.0 and temperature of 40°C. Compared to the wheat gluten, the obtained hydrolysates exhibited enhanced functional attributes, including heightened solubility (43%), increased emulsifying activity (93.08 m^2^/g), and improved radical scavenging properties. Furthermore, these hydrolysates demonstrated enhanced antioxidant potential, as evidenced by elevated ABTS (2,2′-azino-bis-(3-ethylbenzothiazoline-6-sulfonic acid) of 81.25% and DPPH (2,2-diphenyl-1-picrylhydrazyl) of 56.46% radical scavenging activities and also exhibited a higher *α*-amylase inhibitory effect of 33.98%. The enhancement in functional characteristics of wheat gluten hydrolysates was observed by Fourier transform infrared spectroscopy. The percentage of free amino acids obtained by protease-mediated hydrolysates increased significantly compared to the unhydrolyzed wheat, which was observed by high-performance liquid chromatography. These findings suggest that wheat gluten hydrolysates hold promising potential as functional and nutritional food ingredients in the food industry, owing to their enhanced functionalities and potential antioxidant and antidiabetic properties.

## 1. Introduction

Wheat gluten, a major constituent representing around 72% of the total wheat protein content, plays a crucial role in baking due to its indispensable properties such as elasticity, viscosity, and water absorption [1]. Unlike most proteins, gluten proteins possess distinctive and resilient structures that remain intact even under high temperatures [2]. The conventional function of wheat gluten primarily pertains to augmenting the texture of bread. However, its inherent insolubility and viscoelastic properties offer avenues for pioneering food applications via enzymatic or chemical modifications. Previous investigations have primarily focused on three key objectives regarding wheat gluten protein and protease activity. Firstly, researchers aim to understand the correlation between gluten protein physicochemical properties and functional traits, particularly exploring enzymatic modifications to enhance solubility for optimizing protein effectiveness in stabilizing foams and emulsions [3]. The second goal is to identify bioactive peptides, particularly relevant to celiac disease stemming from gluten intolerance, with research directed toward uncovering impactful peptides [4–6]. The third thrust involves deciphering the inherent structural and genetic features of proteins by comparing their constituent peptides, providing valuable insights into the genetic and structural makeup of proteins.

Enzymatic hydrolysis emerges as a sustainable and environmentally friendly approach for enhancing the phytochemistry, functionality, and bioactivity of plant proteins, surpassing chemical methods by reducing the reliance on harsh chemicals and alleviating associated hazards [7–9]. Enzymatic hydrolysis represents a promising approach for reducing the allergenicity of wheat gluten, given that the allergic epitopes of wheat gluten typically comprise five to twenty amino acids long [10]. Several researchers have explored that the enzymatic degradation of gluten offers a viable method to develop foods with diminished allergenicity and improved functional properties [11]. Functional attributes, separate from intact proteins, encompass enhancements in viscosity, solubility, and sensory characteristics, as well as capabilities in retaining oil and water, forming foam, and facilitating emulsification [12, 13].

Hydrolysis heightens plant protein solubility and surface hydrophobicity, unveiling latent hydrophobic clusters. This exposes improved oil-binding affinity, elevating interactions at liquid interfaces, augmenting emulsification, and foaming properties [14]. Remarkably, mild enzymatic treatments utilizing pepsin and papain have demonstrated comparable foamability to egg white protein when applied to gluten, while the addition of adjuvant therapy significantly enhances its emulsifying capacity [15, 16]. Furthermore, studies by Cian et al. [17] have highlighted the outstanding emulsifying properties of gluten peptides with a low degree of hydrolysis (DOH) achieved through the use of trypsin and alcalase. Recent enzymology progress has yielded economical biocatalysts via recombinant microbes, surpassing natural sources. Proteases play a pivotal role in protein breakdown for bacterial assimilation driven by their limited natural availability. When produced inside cells, these enzymes are then covalently linked to cell walls upon release. These breakthroughs usher in versatile enzyme applications across diverse biotechnological domains [18, 19]. Furthermore, studies have highlighted that interactions between peptides and phenolic compounds enhance the antioxidant capacity profile [20, 21]. The International Diabetes Federation's global diabetes report in 2021 revealed that approximately 783 million individuals worldwide are afflicted by diabetes, constituting a global prevalence of 10.5% [15, 16]. A critical aspect of diabetes management involves the use of *α*-amylase and trypsin inhibitors, which impede the digestion of dietary carbohydrates, thereby diminishing the likelihood of postprandial hyperglycemia. Consequently, *α*-amylase inhibition assays serve as a prominent technique for assessing new antidiabetic agents derived from natural sources during drug development [22, 23].

Various fungal and bacterial cultures have been harnessed for the production of protease enzymes, which can be effectively utilized for the hydrolysis of gluten. In this context, the protease producers, protease from *Lactobacillus sanfranciscensis* demonstrated a DOH of 80% on wheat gluten under the following conditions, i.e., pH 7.0, temperature 30°C, and a duration of 32 h [24]. Correspondingly, the protease derived from *A. niger* achieved a DOH of 70% on wheat gluten under conditions of pH 8.0, temperature 40°C, and a duration of 32 h [25]. *Lactobacillus brevis*-derived protease exhibited a DOH of 52% on wheat gluten at pH 6.0, temperature 30°C, and a duration of 24 h [26]. These findings provide valuable insights into the proteolytic activity of various microbial proteases [27] on wheat gluten and highlight their potential for gluten hydrolysis in different enzymatic conditions. *Bacillus* sp. is the most economically utilized protease producer among the many microorganisms.

This investigation is aimed at analyzing the DOH of wheat gluten by optimizing various hydrolysis parameters, such as temperature, time, and pH. Functional properties such as solubility, emulsifying activity, radical scavenging activity, and potent *α*-amylase inhibition have also been observed. Furthermore, Fourier transform infrared spectroscopy was utilized to analyze the functional groups, and high-performance liquid chromatography was used for the quantification of amino acids in wheat gluten hydrolysates.

## 2. Materials and Methods

### 2.1. Chemicals and Microorganisms

*Bacillus subtilis* strain MTCC 2423 was procured from the Microbial Type Culture Collection, India. Wheat gluten powder was purchased from Manidharma Biotech Private Limited, Chennai. All the analytical chemicals were obtained from SRL & HIMEDIA, Bangalore, India, and the deionized water was obtained using a Milli-Q purification system (Millipore, USA).

### 2.2. Protease Production Media

The *Bacillus subtilis* was cultivated in a medium comprising 1% *Casuarina equisetifolia* hydrolysate, 1% soyabean meal hydrolysate, 0.5% peptone, 0.5% casein, 0.5% MgSO_4_.7H_2_O, 0.5% Na_2_CO_3_, 0.5% KH_2_PO_4_, and 0.001% Fe_2_SO_4_.7H_2_O, and the incubation was conducted with an initial pH 8.0 at 37°C [28]. Cultivation took place in a flask volume of 500 mL containing a sterilized fermentation medium (200 mL). All the flasks were incubated with continuous agitation at 150 rpm for 7 days at 37°C, and the experiments were conducted in triplicate. Throughout the fermentation period, flasks were sacrificed daily, followed by centrifugation at 5000 rpm for 30 minutes to eliminate cell debris, and the supernatant was subjected to liquid-liquid extraction. The supernatant was saturated with 60% (*w*/*v*) of ammonium sulfate and *t*-butanol was added in the ratio of 1.0 : 1.0 (*v*/*v*), and the tubes were kept at 30°C for 1 h. The tubes were centrifuged at 2000 g for 10 min, and the three phases formed were collected separately. The enzyme is usually precipitated in the middle layer. To enrich the protease concentration, the interfacial precipitate was collected and subjected to protease assay and wheat gluten hydrolysate production.

### 2.3. Protease Assay

Protease activity was assessed using Sigma's protease activity assay, as described by Marathe et al. [29]. The assay mixture consisted of 1 mL of the culture supernatant to 1 mL of 0.05 M phosphate buffer containing 2% casein, followed by incubation for 10 minutes at 37°C, and subsequently, the reaction was terminated by the addition of 110 mM trichloroacetic acid (5 mL). Furthermore, after 30 minutes of incubation at 37°C, the reaction mixtures were filtered and transferred to new tubes. After that, 5 mL Na_2_CO_3_ and 1 mL Folin's reagent were promptly added, with a final incubation step for 30 minutes at 37°C, followed by filtration again, and the optical density of the filtrates was determined at 660 nm [29].

### 2.4. Production of Enzymatic Hydrolysate of Wheat Gluten

Wheat gluten concentration was prepared by dissolving gluten powder in 100 mL of deionized water for 120 minutes with mild agitation at 60 rpm. Following this, the solution was allowed to hydrate overnight at 4°C to facilitate subsequent sonication. The gluten suspension was then sonicated using a Digital Sonifier 450 (Branson, CT, USA) under the following conditions: operating frequency -25 kHz and maximum output power -200 W in a water–ice bath for 10 min. For the one-factor-at-a-time (OFAT) approach, pH (4.0–8.0), temperature (30–40°C), substrate concentration (1, 5, 10, and 15%), and time (1-6 h) have been varied with 1000 U/mL of partially purified protease as constant. The DOH was subsequently evaluated using the established formol titration method described by Sorensen's formol titration method proposed in the study of Rahaman et al. [30]. To terminate the hydrolysis process, the mixtures underwent a controlled heating phase at 80°C for 10 minutes. Subsequently, centrifugation was executed at 10,000 g and 4°C, followed by the careful collection and storage of the resulting supernatant for future experimental purposes.

### 2.5. Determination of Functional Properties of Wheat Gluten Hydrolysate

#### 2.5.1. Solubility

Protein suspensions were prepared at a concentration of 1% using a 0.01 M phosphate buffer solution. The pH of the suspensions was adjusted to different values (4.0, 5.0, 6.0, 7.0, and 8.0) by adding either 1 M NaOH or 1 M HCl. To ensure complete hydration, the suspensions were mixed for 30 minutes at 30°C using a mechanical stirrer. Following this, the suspensions were subjected to centrifugation for 10 minutes at 10,000 rpm and 4°C. The pH value at which the protein exhibited maximum solubility was determined, and the protein content in the resulting supernatant was quantified using the Lowry method, as outlined in previous studies [31].

#### 2.5.2. Emulsion Activity and Emulsion Stability Index

The emulsion activity index (EAI) was assessed according to the method described by Chatterjee et al. [32] and Deng et al. [33]. In a 50 mL centrifuge tube, a combination of castor oil and protein hydrolysate was homogenized for 10 minutes to form an emulsion. Aliquots were collected from the lower portion of the reaction tube, and the emulsion was diluted with 0.1% sodium dodecyl sulfate (SDS) solution. The absorbance of the diluted emulsion was measured at a wavelength of 500 nm using a blank solution of 0.1% SDS. The emulsions were left undisturbed at room temperature for 10 minutes. Then, 100 *μ*l samples were taken from the bottom of the beaker and diluted with 5 ml of 0.1% SDS solution, and the absorbance was measured at 500 nm by spectrophotometer to determine the EAI and emulsion stability index (ESI). All experiments were performed in triplicate to ensure precision and reproducibility.

#### 2.5.3. Degree of Hydrolysis

The DOH was evaluated using a modified version of Sorensen's formol titration technique. A 3 mL solution of the 1% sample was added into formalin solution (4 mL), and a few drops of phenolphthalein indicator and 0.1 M NaOH were used for titration until the suspension turned pink. The NaOH volume required to achieve the colour change was recorded and utilized to determine the degree of hydrolysis. To ensure accuracy, all experiments were conducted in triplicate. The DOH (%) was measured using Eq. (1) corresponding to NaOH volume consumed, following the methodology described by Rahaman et al. [30].

(1) $$\begin{array}{c} \text{DOH }\left(\%\right)=\frac{C_{1}\times \left(V_{1}-V_{2}\right)\times 1000}{C_{2}\times V\times h_{\text{tot}}}\times 100, \end{array}$$

where *C*_1_ denotes concentration of NaOH (mol/L), *V*_1_  denotes the volume of NaOH consumed by hydrolyzed wheat gluten (mL), *V*_2_ denotes the volume of NaOH consumed by the unhydrolyzed wheat gluten (mL), *C*_2_ represents the concentration of the sample (g/L), *V* represents the volume of sample (mL), and *h*_tot_ represents the total number of peptide bonds in the protein substrate (8.38 meqv/g gluten protein).

#### 2.5.4. ABTS Radical Scavenging Activity

This experiment measures the ABTS radical peroxidation inhibition percentage, which appears as a shift in colour from blue to green at 734 nanometers. The procedure, adapted from Arts et al. [34], involved preparing ABTS•+ by dissolving potassium persulfate (2.45 mM) and ABTS (7 mM) in phosphate-buffered saline (PBS) at pH 7.4 to create ABTS•+. The solution was incubated for 16 hours without light to facilitate the formation of the radical cation. Subsequently, the ABTS•+ stock solution was mixed with PBS buffer and equilibrated to 30°C, and its optical density was adjusted to 0.7 ± 0.02 at 734 nm using a UV-Vis spectrophotometer (Cary 60, Agilent Technologies, USA).

Ascorbic acid, dissolved in 80% ethanol, served as a positive control. Briefly, 200 *μ*L of each sample was mixed with 2 mL of the equilibrated ABTS•+ solution, and the decrease in optical density was monitored over five minutes. Blank controls were included for each sample, and the radical scavenging capacity of the samples was then compared to a standard curve prepared using ascorbic acid solutions ranging from 6.25 to 200 *μ*M. The results were expressed as milligrams of ascorbic acid equivalent per gram of sample on a protein basis. The percentage inhibition of ABTS•+ scavenging was determined using the following:

(2) $$\begin{array}{c} \text{ABTS scavenged }\left(\%\right)=\frac{A_{\text{i}}-A_{\text{f}}}{A_{\text{i}}}\times 100, \end{array}$$

where *A*_f_ indicates the final absorbance of the sample and *A*_i_ indicates the initial absorbance of the sample.

#### 2.5.5. DPPH Radical Scavenging Activity

The DPPH radical scavenging activity of SPH and its fractions was assessed following a method adapted from Girgih et al. [35], with minor adjustments. For this experiment, DPPH was prepared by diluting it in methanol at a concentration of 0.1 mM. A 1 mL aliquot of each sample and 1 mL of the DPPH radical solution were mixed gently in a test tube, and the mixture was vortexed for 1 minute. Afterwards, the mixture was kept at room temperature for half an hour in the dark condition. The buffer was used in the blank test, and ascorbic acid was used as the standard. The absorbance of the reaction mixture was estimated at 517 nm using a UV-Vis Spectrophotometer, and the DPPH radical scavenging activity percentage was determined using

(3) $$\begin{array}{c} \text{DPPH radical scavenging activity }\left(\%\right)=\frac{A_{\text{b}}-A_{\text{s}}}{A_{\text{b}}}\times 100, \end{array}$$

where *A*_s_ and *A*_b_ are absorbance of the sample and blank, respectively.

#### 2.5.6. *α*-Amylase Inhibition Assay

The sample's *α*-amylase inhibitory activity was assessed based on a modified version of the method by Kumar et al. [36]. Briefly, 500 *μ*L of the sample solution in a phosphate buffer (pH 6.9, 0.006 M NaCl) was incubated with 250 *μ*L of enzyme solution (0.5 mg/mL) for 10 minutes at 25°C ± 1°C. Subsequently, 500 *μ*L starch solution (0.5% *w*/*v*) prepared in 0.02 M phosphate buffer at pH 6.9 was added and incubated for an additional 10 minutes at the same temperature. The reaction was terminated by the addition of 1 mL DNS reagent. Further, the mixture was heated at 90°C for 10 minutes and the measurement was done at the absorbance of 540 nm and the absorbance was measured at 540 nm using a UV-Vis Spectrophotometer. Untreated enzymes served as a control, and individual samples were compared to reference points established beforehand. Acarbose was employed as a standard enzyme inhibitor. The experiment was performed three times for each sample to ensure reproducibility, and the *a*-amylase inhibition (%) was calculated by the following:

(4) $$\begin{array}{c} \alpha ‐\text{Amylase inhibition }\left(\%\right)=\frac{A_{\text{b}}-A_{\text{s}}}{A_{\text{b}}}\times 100, \end{array}$$

where *A*_b_ represents the optical density of the blank control containing only the phosphate buffer solution and *A*_s_ represents the optical density of the sample solution after the enzymatic reaction.

#### 2.5.7. FTIR Spectra

Fourier transform infrared (FTIR) spectroscopy (Spectrum RXI, PerkinElmer) was employed to analyze changes in the secondary structure of the protein samples before and after hydrolysis. Spectra were acquired in the midinfrared region (4000-400 cm^−1^) with a resolution of 4 cm^−1^. The amide I band (1600-1700 cm^−1^), which is sensitive to protein secondary configuration, was analyzed using the established peak deconvolution methods described by Susi and Byler [37]. This method involves fitting Gaussian peaks to the amide I band and assigning them to specific secondary structure components. Quantitative analysis of the secondary structure composition was performed using the same method. All FTIR experiments were conducted in duplicate to ensure reproducibility.

#### 2.5.8. Amino Acid Analysis

The amino acid composition of the freeze-dried proteins was determined using acid hydrolysis followed by analysis with high-performance liquid chromatography (HPLC, Agilent Technologies, USA) equipped with a PicoTag column. The flow rate was adjusted to 0.5 mL/min, and a variable wavelength detector at a wavelength of 338 nm was used. The hydrolysis step involved incubating the protein samples with 6 N HCl at 110°C for 24 hours. Subsequently, the resulting hydrolyzed solutions underwent neutralization through the addition of 4 M NaOH. After neutralization, the samples were centrifuged at 10,000 g for 10 minutes. The collected supernatants from this process were then utilized for amino acid analysis under the temperature of 38°C.

## 3. Results and Discussion

### 3.1. Properties of *Bacillus subtilis* Protease

A maximum protease activity of 7.2 U/mL was obtained from *Bacillus subtilis* MTCC 2423. The produced protease was partially purified using three-phase partitioning with an activity recovery of 81% with 21.8-fold purification. From Table 1, the optimum pH and temperature of protease were 6.0 and 40°C. Upon increasing the pH conditions to 9.0, a drastic decrease in the protease activity was observed. The kinetic characteristics, including *K*_cat_, *K*_m_, and *K*_cat_/*k*_m_ of the enzyme were established by assessing the protease activity. The *K*_cat_, *K*_m_, and *K*_cat_/*k*_m_ values for protease are 1.11 mM, 27.31 min^−1^, and 24.31 min/mM exhibiting higher substrate affinity and catalytic efficiency. The thermostability of the crude protease was evaluated by measuring its half-life at temperatures ranging from 40°C to 50°C. Initially, at 40°C, the half-life of protease was found to be 784.2 minutes.

When the temperature was increased to 50°C, protease showed a half-life of around 289.14 minutes. Protease enzymes demonstrate specific activity patterns influenced by particular conditions, with their effectiveness typically declining beyond optimal conditions. The enzyme's stability relies on its optimal pH range, while more extreme pH values cause irreversible damage to its tertiary structure in a time-dependent manner; further, it deviates from the optimum pH, faster inactivation, leading to hindrance in activity, and distorted shape, reduced reaction rates, and loss of catalytic function [38].

### 3.2. Optimization of Enzymatic Hydrolysis of Wheat Gluten

The enzyme-mediated hydrolysis of gluten protein involves two primary steps: pretreating the gluten solution through ultrasonication, followed by proteolytic treatment of the gluten. Utilizing the framework laid out by Deng et al. [33], this study delves into the enzymatic degradation of wheat gluten protein employing a *Bacillus subtilis* protease. The overarching objective is to optimize the sonication-assisted enzymatic breakdown process to produce amino acid hydrolysates from gluten. Regarding pH, the optimal DOH was obtained at pH 8.0 resulting in the range of 21.14% to 34.07%. Regarding pH, a considerable increase in DOH was attained when the protease treated the gluten at pH 7.0, and the higher protease activity and the better reactivity with gluten at pH 8.0 are likely to enhance the DOH to 14.3%. Conversely, in aqueous solution at acidic pH 4.0–5.0 and higher conditions of pH 9.0, the DOH of gluten protein was low.

An increase in the temperature conditions (40°C) from room temperature of enzymatic treatment of wheat gluten led to an increase in the DOH (21.14%). This increase is likely attributed to the enzyme's optimal temperature range and its stability under these conditions, and it was observed that with further escalation of temperature conditions during enzymatic hydrolysis to 50 and 60°C, the DOH was decreased. These findings corroborate previous observations by Djekrif et al. [27] and Rashmi et al. [39], who reported a similar decrease in protease activity at higher temperatures. This phenomenon might be attributed to the adverse effect of high temperatures on the accessibility of the enzyme's catalytic sites to the wheat gluten substrate, as suggested by the positive correlation between good hydrolysis and adequate substrate exposure reported by Singh et al. [40].

A wheat gluten concentration of 5% was chosen as the standard, as higher concentrations (10% and 15%) resulted in decreased DOH (27.2% and 14.9%, respectively). In order to determine the optimal hydrolysis time for achieving the highest degree of hydrolysis (DOH) of gluten, various incubation periods were assessed. It has been established that a very short hydrolysis time (less than 30 minutes) could result in insufficient breakdown of proteins and subsequently, lower DOH levels. Therefore, identifying the optimal enzymatic hydrolysis duration is critical to maximizing DOH. Since minimal differences were observed at (1-6 h), the shortest incubation time was selected to expedite the hydrolytic process for gluten hydrolysate preparation. It was noted that the DOH increased with the optimization of hydrolysis duration aligning with the conclusions drawn by Yang et al. [41]. Similarly, the DOH showed an initial increase and subsequent decrease in the hydrolysates. From the OFAT approach, the highest DOH (34.07%) was achieved at a hydrolysis time of 2 h for 5% gluten protein, with an enzyme amount of 1000 units, a temperature of 40°C, and a pH of 8.0. The preliminary enzymatic treatment of wheat gluten protein (Table 2) provided important information regarding each parameter of enzymatic hydrolysis.

### 3.3. Functional Characteristics of Gluten Hydrolysates

#### 3.3.1. Solubility

**Figure 1:**
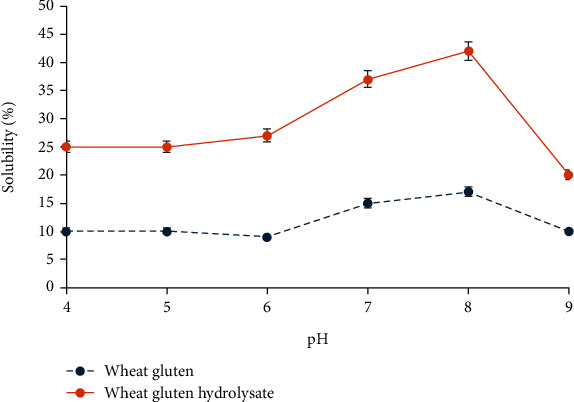
Solubility of wheat gluten and wheat gluten hydrolysate with respect to pH.

During the hydrolysis process, there was a significant increase in the water solubility of gluten, as demonstrated in Figure 1. The solubility of natural wheat gluten exhibited a bell-shaped curve pattern, which is commonly observed and influenced by pH. The solubility of wheat gluten exhibits a gradual increase beyond pH 6, consistent with findings from a prior study by Olanca and Özay [42], which noted the protein solubility diminished near pH 6, closely aligning with the isoelectric point of gluten proteins. Consistent with the findings of Banerjee et al. [43], protein aggregation is facilitated at the isoelectric point (pI) owing to the neutralization of charge repulsion between protein molecules, leading to increased hydrophobic interactions. This phenomenon explains the observed higher solubility of gluten under alkaline conditions compared to acidic ones. Alkaline environments generally promote protein dissociation and disaggregation, consequently enhancing protein solubility. Previous studies have suggested two factors contributing to the improved solubility of protein hydrolysates in comparison to the raw protein. Primarily, it is attributed to the reduction in the protein's secondary structure. Secondly, the enzymatic action leads to the liberation of smaller peptides/polypeptides from the protein, which enhances the solubility of the hydrolysate. Previous studies have demonstrated that wheat gluten solubility has increased from 14% to over 60% after hydrolysis [44]. The process of enzymatic hydrolysis substantially reduces the average molecular mass of proteins, liberating ionizable groups and thereby augmenting protein solubility. Multiple investigations have provided evidence that enzymatic hydrolysis of plant proteins leads to a significant enhancement in solubility across various pH ranges [45].

#### 3.3.2. Emulsion Activity Index

**Figure 2:**
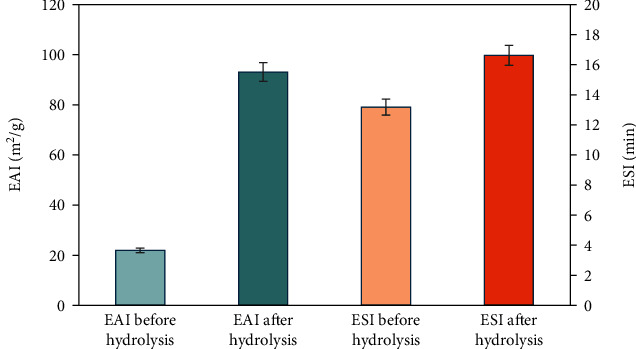
Emulsifying activity index and emulsion stability index of wheat gluten before and after hydrolysis by *Bacillus* protease.

The emulsifying activity of wheat gluten hydrolysate demonstrated a significant increase from 22 to 93.08 m^2^/g, as depicted in Figure 2. However, the emulsifying stability did not exhibit a notable change. This observation suggests a lack of correlation between these functional characteristics. The interaction between peptides and lipids may be enhanced during the expansion of wheat gluten and the enzymatic breakdown of peptides, resulting in reduced interfacial tension and promoting interaction between peptides and lipids thus increasing emulsifying activity [46, 47]. However, smaller peptides generated by enzymatic hydrolysis may exhibit decreased emulsifying stability. These smaller peptides may not form generate films of adequate thickness essential for sustaining emulsions.

Wang et al. [48] documented that wheat gluten hydrolysates, particularly the 50 kDa fraction, exhibited a twofold increase in emulsifying activity index compared to native gluten. Similarly, Deng et al. [33] observed a significant enhancement in emulsifying activity (18.05 m^2^/g to 58.65 m^2^/g) following gluten hydrolysis, although emulsion stability remained largely unchanged. Similarly, emulsification activity index (EAI) following protein hydrolysis has been reported in various sources such as corn, lupin, soy, rice bran, and potato. This observation suggests that the heightened presence of hydrophobic groups in protein hydrolysates contributes to the enhancement of emulsifying properties [45].

#### 3.3.3. Scavenging Analysis

**Figure 3:**
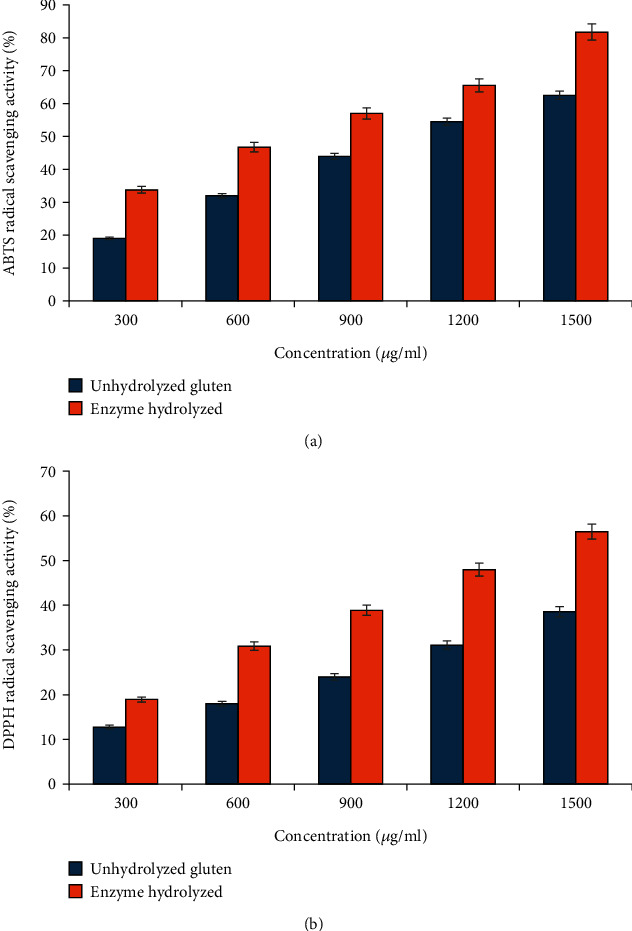
(a) ABTS and (b) DPPH radical scavenging activity of wheat gluten before and after hydrolysis by *Bacillus* protease.

The results from the ABTS and DPPH radical scavenging analyses conducted on wheat gluten and its hydrolysates, using proteases derived from *Bacillus subtilis*, are depicted in Figure 3. Notably, the antioxidant activity exhibited an augmentation following protease-driven hydrolysis, with wheat gluten hydrolysates displaying superior antioxidant potential compared to the unhydrolyzed wheat gluten. The employment of enzymes, particularly those derived from *B. subtilis*, seemed to bolster the antioxidant attributes of the gluten, evidenced by a surge in ABTS radical scavenging activity from 62.5 ± 0.25% to 81.25 ± 0.22% post-hydrolysis by *B. subtilis*-derived protease. The IC_50_ values for the hydrolysate and wheat gluten were measured at 717.36 ± 0.26 and 1108.21 ± 0.2 *μ*g/mL, respectively. A corresponding enhancement was observed in the DPPH radical scavenging activity, with the wheat gluten hydrolysate showcasing an efficiency of 56.46 ± 0.32% compared to 38.56 ± 0.24% in unhydrolyzed form. The IC_50_ values for the hydrolysate and wheat gluten were measured at 1271 ± 0.28 and 2061.47 ± 0.43 *μ*g/mL. These observations suggest the generation of proteins with simplified structures and reduced molecular weights through proteolytic action, thereby bolstering the antioxidant capacity. This phenomenon is attributed to the efficacy of *B. subtilis*-derived protease in cleaving peptide bonds.

Nonetheless, ascorbic acid exhibited markedly superior DPPH and ABTS radical scavenging activities compared to the hydrolysates, even at lower concentrations. It has been reported that the radical scavenging properties of hydrolysates are determined by the types of amino acids liberated during the hydrolysis process. Due to their role as prominent proton donors in oxidative reactions, imidazole rings, along with hydrophobic amino acids, exhibit significant radical scavenging properties [49]. Cian et al. [17] explored the relationship between hydrophobicity, molecular weight, and antioxidant activity of wheat gluten hydrolysates digested with protamex enzyme. Their findings revealed that peptides with hydrophobicity and moderate molecular weight demonstrated the most potent ABTS radical scavenging activity. Consistent with these findings, Seyedain-Ardabili and Azizi [50] reported analogous results in their study on wheat gluten protein hydrolyzed with ficin. Their research revealed that the ABTS radical scavenging activity method exhibited higher antioxidant activity values compared to the DPPH radical scavenging activity method. These outcomes align with prior studies suggesting that hydrolysis can effectively enhance antioxidant activity.

#### 3.3.4. Antidiabetic Assay

**Figure 4:**
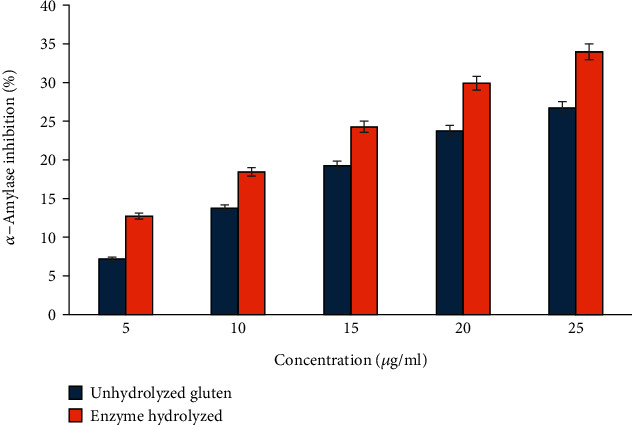
Inhibition of *α*-amylase activity of wheat gluten before and after hydrolysis by *Bacillus* protease.

The *α*-amylase inhibitory activity of the hydrolysates was assessed to investigate their potential antidiabetic effects (Figure 4). Both acarbose and hydrolysates derived from *B. subtilis*-mediated protein hydrolysis demonstrated inhibitory effects on the enzyme. Wheat gluten hydrolysate showed a higher inhibition of 33.98 ± 0.18%, while the unhydrolyzed wheat gluten showed an inhibition of 26.73 ± 0.3%. IC_50_ values for the hydrolysate and wheat gluten were measured at 39.18 ± 0.32 and 47.45 ± 0.28 mg/mL, respectively. IC_50_ values of the hydrolysates against *α*-amylase were notably higher compared to acarbose. At an equivalent dosage, acarbose exhibited markedly greater inhibition of *α*-amylase compared to the raw hydrolysates. However, acarbose is associated with several adverse effects, highlighting the potential of natural hydrolysates as a safer alternative [51].

As per findings from Bozkurt et al.'s investigation [52], there existed a significant correlation between the DOH by the enzymes and the resulting IC_50_ values, with hydrolysates possessing higher DOH exhibiting lower IC_50_ values. The antidiabetic effects of the peptides are indicated by factors such as their size, sequence, and the inclusion of hydrophobic amino acids within the peptide structure. Bioactive peptides possess the capacity to diminish or hinder substrate binding by engaging with the active site of an enzyme. Alternatively, they can interact with allosteric sites, leading to conformational changes that reduce substrate affinity. Additionally, peptides might target ion-binding sites, inducing unstable enzyme conformations that disrupt substrate interaction [53]. Consequently, a higher DOH results in the generation of a larger quantity of peptides, thereby enhancing the inhibition of the enzyme [54].

#### 3.3.5. FTIR Analysis

**Figure 5:**
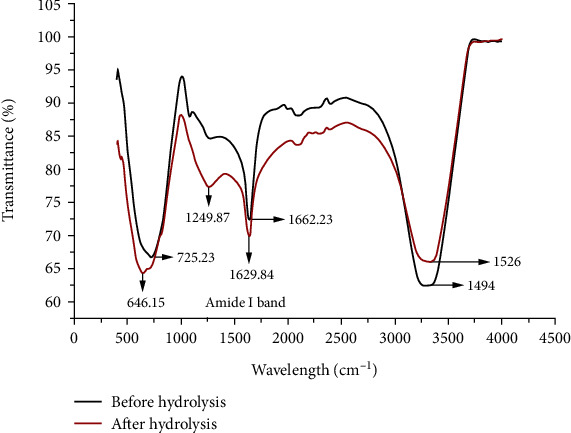
FTIR analysis before and after hydrolysis of gluten.

FTIR spectroscopy is a valuable tool for investigating the secondary structure and specific amino acids present in proteins. The configuration of proteins is crucial in shaping gluten network structures [55]. FTIR analysis was employed to detect changes in functional groups associated with the hydrolysis process, building upon previous investigations by Cian et al. [17], Cui et al. [56], and Sun et al. [15, 16]. The amide I band, situated within 1600-1700 cm^−1^, is highly sensitive to alterations in secondary structure and has been extensively studied to understand the structure-spectrum relationship. In this investigation, the FTIR spectra of amide I region underwent analysis, and curve fitting procedures were executed to discern the amide I bands present in both wheat gluten and its hydrolysate, as illustrated in Figure 5.

Additionally, the functional group components were identified. Amide I band assignments have been reported in the literature to occur between 1600 and 1700 cm^−1^ [57]. According to earlier studies, specific secondary structures were identified within defined wavenumber ranges: *β*-sheet (1623–1641 cm^−1^), *α*-helix (1648–1667 cm^−1^), and turns (1670–1687 cm^−1^) [33]. Notably, the amide I band at approximately 1662.23 cm^−1^ conventionally signifies the presence of *α*-helical structures [17]. After hydrolysis, a discernible shift in the *α*-helix band was obtained, transitioning from 1651 cm^−1^ to 1658 cm^−1^. Furthermore, shifts at distinct wavenumbers, such as 1494 cm^−1^ (C-H bonding), 1526 cm^−1^ (N-O stretching), 1249.87 cm^−1^ (C-O stretching), and 725.23 cm^−1^ (C-H bending), provide evidence of altered chemical bonding configurations [58].

The enzymatic cleavage and structural rearrangement of wheat gluten, induced by the fragmentation of peptide chains and the disturbance of higher molecular weight peptide sequences, result in the release of amino acids [30]. This phenomenon is believed to underpin the transition from *α*-helical to *β*-sheet structures. Importantly, this transformation is facilitated by the unfolding of the original globular gluten structure, concurrent with a reduction in turn ratio and the promotion of elongated *β*-sheet chains [59].

#### 3.3.6. Determination of Amino Acid Composition

**Figure 6:**
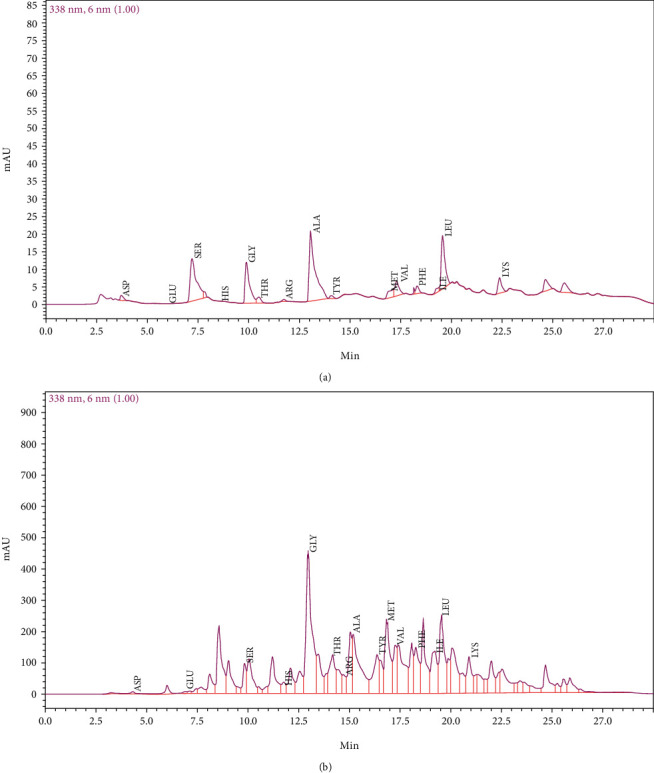
HPLC chromatogram of (a) wheat gluten and (b) wheat gluten hydrolyzed by *Bacillus*-derived protease.

Wheat gluten proteins are recognized as valuable reservoirs of amino acids in the human diet. The amino acid composition (mg per 100 g protein solution) of wheat gluten hydrolysate is shown in Table 3. The World Health Organization and the Food and Agriculture Organization have established dietary protein recommendations, indicating that nine essential amino acids, namely, phenylalanine, isoleucine, leucine, histidine, lysine, methionine, threonine, valine, and tryptophan, are necessary to maintain normal bodily functions [60]. The free amino acid chromatogram of wheat gluten is shown in Figure 6. Glycine was the most abundant amino acid, followed by leucine and methionine. The percentage of free amino acids obtained by protease-hydrolyzed wheat gluten was increased significantly compared to the unhydrolyzed wheat gluten, indicating a successful hydrolysis process for most amino acids. Thus, soluble protein composites are highly promising as a primary protein source for adults due to their easy digestibility, versatile nature, and balanced amino acid profile.

## 4. Conclusion

This study highlights the potential of utilizing *Bacillus subtilis* MTCC 2423 protease for the hydrolysis of wheat gluten protein, resulting in the production of hydrolysates with enhanced functional properties, achieved under optimized conditions involving a 2-hour hydrolysis time for 5% gluten protein, enzyme amount of 1000 units, a temperature of 40°C, and a pH of 8.0. The optimized enzymatic hydrolysis conditions resulted in significant improvements in solubility, with emulsifying activity measuring at 93.08 m^2^/g, and antioxidant potential at 81.25% observed in the wheat gluten hydrolysates. These findings contribute to the ongoing efforts in the food industry to explore sustainable ways of enhancing the nutritional and functional qualities of food ingredients. The demonstrated antioxidant and *α*-amylase inhibitory effects further suggest the potential of wheat gluten hydrolysates as functional ingredients with health-promoting benefits. Overall, this study underscores the feasibility and potential of utilizing wheat gluten hydrolysates as valuable additives in the development of functional foods with improved nutritional profiles and bioactive properties.

## Figures and Tables

**Table 1:** Properties of *Bacillus subtilis* protease.

Parameter	Protease
Optimum pH	8.0
Optimum temperature (°C)	40
*K*_m_ (mM)	1.114 ± 0.231
*K*_cat_ (min^−1^)	27.31 ± 2.14
*K*_cat_/*K*_m_ (min^−1^/mM^−1^)	24.51 ± 2.7
*E*_a_ (kJ·M^−1^)	1.37 ± 0.14
*t*_1/2_ (min) at 40°C	784.2 ± 37.2
*t*_1/2_ (min) at 50°C	291.8 ± 12.33

**Table 2:** Classical OFAT approach for the optimization of conditions of *Bacillus subtilis* protease-treated wheat gluten.

pH	Temperature (°C)	Substrate concentration (%)	Time (h)	Degree of hydrolysis (%)
4	30	1	1	0.76
5	30	1	1	2.2
6	30	1	1	4.63
7	30	1	1	13.07
8	30	1	1	14.91
9	30	1	1	1.32
8	40	1	1	21.14
8	50	1	1	17.62
8	60	1	1	16.74
8	40	5	1	29.07
8	40	10	1	27.26
8	40	15	1	14.97
8	40	5	2	34.07
8	40	5	3	28.26
8	40	5	4	27.97
8	40	5	5	27.57
8	40	5	6	27.57

**Table 3:** Amino acid composition (mg per 100 g protein solution) of wheat gluten hydrolysate.

Amino acid	Gluten hydrolysate (mg/100 g)	% free amino acid against TFAA
Lysine	14.6	4.63
Leucine	145.27	9.72
Isoleucine	47	5.91
Methionine	137.35	9.59
Valine	54.1	7.16
Cysteine	17.2	1.17
Tyrosine	24.9	1.58
Threonine	14.4	7.12
Histidine	30.2	3.97
Glycine	358.32	15.6
Proline	15.8	1.24
Alanine	39.2	8.29
Arginine	54.78	8.52
Glutamine	43.56	2.09
Asparagine	54.6	1.607
Serine	64.2	5.28
Phenyl alanine	38.3	9.31
Tryptophan	20.76	1.2

## Data Availability

The data are available upon request.
